# Atorvastatin reduces recurrent decompensation events in advanced cirrhosis in a randomized placebo-controlled trial

**DOI:** 10.1038/s41598-026-41326-4

**Published:** 2026-03-21

**Authors:** Khadija A.M. Glal, Sahar M. El-Haggar, Sherief M. Abdel-Salam, Tarek M. Mostafa

**Affiliations:** 1 Department of Clinical Pharmacy, Faculty of Pharmacy, Assistant lecturer of Clinical Pharmacy, Tanta University, Tanta, 31527 Egypt; 2 Department of Clinical Pharmacy, Faculty of Pharmacy, Tanta University, Tanta, 31527 Egypt; 3 Tropical Medicine and Infectious Diseases, Faculty of Medicine, Tanta University, Tanta, 35127 Egypt

**Keywords:** Atorvastatin, Liver Cirrhosis, Hepatorenal Syndrome, Oxidative Stress, Bacterial Translocation, Diseases, Gastroenterology, Medical research

## Abstract

Statins exhibit pleiotropic anti-inflammatory and antifibrotic properties that may attenuate the progression of cirrhosis. This study aimed to evaluate the efficacy and safety of atorvastatin in preventing recurrent decompensation events (DDs) and in modulating the gut–liver axis among patients with decompensated cirrhosis. In this randomized, double -blind, placebo-controlled trial, 100 adults with decompensated cirrhosis were randomly assigned in a 1:1 ratio to receive either atorvastatin (20 mg/day) or placebo for six months. The primary endpoint was the incidence of recurrent decompensation events (DDs). Secondary outcomes included changes in biomarkers of oxidative stress (malondialdehyde [MDA]), systemic inflammation (nuclear factor kappa B [NF-κB], C-reactive protein [CRP], and erythrocyte sedimentation rate [ESR]), intestinal permeability (zonulin), and endotoxemia (lipopolysaccharide [LPS]). Atorvastatin treatment significantly reduced the cumulative recurrence of cirrhosis-related complications compared to placebo (36% vs. 72%; HR 0.50; 95% CI, 0.33–0.75; *P* < 0.001). Notably, atorvastatin conferred complete protection against hepatorenal syndrome (HRS) (0% vs. 20% in the placebo group; all Type 2). These clinical improvements were mirrored by significant reductions in MDA, NF-κB, LPS, and zonulin levels (*P* < 0.05), indicating a robust attenuation of systemic inflammation and restoration of intestinal barrier integrity. Atorvastatin was well-tolerated; while mild myalgia was more frequent in the intervention group (14% vs. 2%), elevations in transaminases were transient and clinically insignificant. In this randomized trial, atorvastatin was associated with a lower recurrence of decompensation events and a reduced incidence of hepatorenal syndrome compared with placebo. These effects were accompanied by improvements in biomarkers related to systemic inflammation and gut–liver axis dysfunction. Atorvastatin was generally well tolerated, although mild myalgia occurred more frequently and warrants clinical monitoring. While these findings suggest a potential role for atorvastatin as an adjunctive therapy in decompensated cirrhosis, confirmation in larger, multicenter studies is required before broader clinical application. **Clinical trial number**: NCT05563389 (https://register.clinicaltrials.gov/prs/beta/studies/S000CHKM00000052/recordSummary).

**Supplementary Information:**

The online version contains supplementary material available at 10.1038/s41598-026-41326-4.

## Introduction

Liver cirrhosis is a progressive and irreversible condition characterized by hepatocellular necrosis and fibrogenesis^1^. The resulting impairment of hepatic function, coupled with portal hypertension (PH), predisposes affected individuals to a spectrum of complications, including ascites, spontaneous bacterial peritonitis (SBP), hepatic encephalopathy (HE), jaundice, variceal bleeding (VB), and hepatorenal syndrome (HRS), ultimately leading to clinical decompensation^2^..

Statins exhibit a wide range of pleiotropic effects extending beyond lipid lowering, including improvement of endothelial dysfunction and demonstrable antioxidant, antifibrotic, anti-inflammatory, antiproliferative, antiangiogenic, proapoptotic, and immunomodulatory properties^3^. Preclinical investigations and selected clinical studies have shown that statins can reduce the hepatic venous pressure gradient (HVPG), enhance hepatic perfusion, and lower the risk of variceal haemorrhage in patients with cirrhosis^4,5^..

Despite advances in medical care, current therapeutic options for decompensated cirrhosis remain limited, and the occurrence or progression of complications continues to carry a substantial mortality risk^6^. Accordingly, the identification of novel therapeutic interventions targeting the pathophysiological mechanisms of hepatic decompensation is urgently needed. Based on this rationale, we conducted a randomized, placebo-controlled clinical trial to assess the efficacy of six months of atorvastatin therapy in preventing the progression of cirrhosis-related complications.

## Patients and methods

### Study design and registration

This randomized, double -blind, placebo-controlled trial was conducted at the Tropical Medicine & Infectious Diseases Department, Tanta University Hospital, Egypt, between June 2022 and February 2023. The study was registered at ClinicalTrials.gov (Identifier: NCT05563389) on June 29, 2022. Ethical approval was obtained from the Research Ethics Committee (Institutional Review Board) of Tanta University (Approval No. 13/6/2022; Protocol Version 1.0, dated June 13, 2022).

All study procedures adhered to the principles of the 1975 Declaration of Helsinki and conformed to CONSORT and ICH Good Clinical Practice guidelines. Written informed consent was obtained from each participant before enrolment, and confidentiality and voluntary participation were ensured throughout the study period.

### Study patients: Inclusion and exclusion criteria

Eligible participants were adults aged 18–75 years of either sex diagnosed with decompensated liver cirrhosis based on standard clinical, laboratory, and imaging criteria. Patients were screened during hospitalization for a decompensation episode. Randomization was performed only after clinical stabilization and resolution of the acute episode, and the study intervention was initiated as secondary prophylaxis to prevent recurrence.

Decompensation was defined as a previous episode of any major cirrhosis-related complication, including ascites, SBP, VB, jaundice, HRS, or HE.

Exclusion criteria included chronic kidney disease G5 as defined according to the KDIGO classification^7^, known statin intolerance or allergy, severe extrahepatic comorbidities such as heart failure NYHA functional class IV and/or ACC/AHA stage D^8^, end-stage respiratory disease GOLD 4, and mMRC grade 3–4^9^, liver transplantation, pregnancy, or lactation were also excluded. Severe extrahepatic comorbidities were excluded to minimize confounding and ensure that clinical outcomes would primarily reflect the effect of the study intervention in decompensated cirrhosis.

Although the original study protocol published on ClinicalTrials.gov (Identifier: NCT05563389) specified narrower eligibility criteria, the exclusion criteria were broadened during patient recruitment to enhance feasibility and improve generalizability. These modifications were approved by the ethics committee before implementation. These changes did not affect the primary endpoint or study design.

### Randomization and protocol procedures

Following screening, eligible participants were randomized in a 1:1 ratio to receive either atorvastatin or placebo using a computer-generated randomization sequence prepared by an independent pharmacist, following CONSORT and ICH Good Clinical Practice guidelines recommendations. Patients in the intervention arm received atorvastatin 20 mg once daily for six months, a dose supported by prior clinical evidence^10,11^. All participants continued their baseline standard-of-care therapies, including diuretics, non-selective beta-blockers, rifaximin, and lactulose, without any dose modification during the study. Allocation concealment was ensured using identical, sequentially numbered medication containers. Investigators, participants, outcome assessors, and data analysts remained blinded to treatment allocation. Adherence was monitored at each follow-up visit through pill counts and structured participant interviews.

### Management of decompensation during the study

Episodes of decompensation events (DDs) were managed according to established international guidelines without altering chronic background therapy. Tense ascites was treated with large-volume paracentesis; HE episodes were managed by temporary adjustment of lactulose and rifaximin doses; and VB was treated using standard endoscopic and pharmacologic measures. This uniform management ensured that observed differences in the recurrence of decompensation events could be attributed to the study intervention rather than to variations in supportive care. Antiviral therapy for chronic hepatitis B virus infection was continued, while hepatitis C virus infection had been fully eradicated in all affected participants. Albumin administration was permitted without restriction.

### Treatment discontinuation and safety monitoring

Treatment discontinuation criteria were prospectively defined and included severe adverse events, clinically significant elevations in liver enzymes (≥ 3× the upper limit of normal), or withdrawal of informed consent. Participant adherence was supported through scheduled monthly clinic visits and biweekly telephone follow-up. All adverse events were continuously monitored, systematically recorded, and independently evaluated by an appointed safety monitor for the duration of the study.

### Blinding (masking)

A strict double -blind design was implemented to minimize performance and detection bias. Participants, treating physicians, research staff, care providers, and outcome assessors remained fully blinded to treatment allocation throughout the study. Atorvastatin and placebo were manufactured in identical-looking pre-filled capsules, matched in color, size, and weight, and dispensed in opaque, tamper-proof containers. This ensured that neither participants nor staff could distinguish between treatment arms based on appearance, packaging, or handling characteristics.

An independent pharmacist, who was not involved in patient assessment or follow-up, generated and maintained the allocation sequence. Each participant was assigned a unique randomization code stored in a secure file accessible only to the pharmacist. This individualized coding system prevented complete unblinding of the cohort if a single participant’s treatment identity needed to be revealed for safety reasons.

Medication logs were kept for each participant, documenting dispensing dates, returned capsules, and adherence checks. Any discrepancies were reconciled by an independent monitor.

Data management followed a multi-layered quality assurance process. Electronic case report forms (eCRFs) were password-protected and stored on encrypted devices. A double-entry system was employed, in which two independent data clerks entered all study data separately, and discrepancies were automatically flagged and resolved by the data management team. Range limits, internal consistency checks, and real-time error alerts were incorporated to ensure accuracy and completeness of entered data.

Data cleaning procedures included cross-checking CRFs with source documents, verifying laboratory values against standard reference ranges, and validating dates, visiting windows, and outcome definitions. Only after all discrepancies were resolved and the database was formally locked did the study team proceed to unblinding and statistical analysis.

Unblinding was permitted exclusively when clinically indispensable, for example, in the setting of a serious adverse event, life-threatening condition, or when treatment knowledge was essential to guide emergency medical decisions. Requests for unblinding were reviewed by the principal investigator and safety monitor and documented in detail.

Participant confidentiality was rigorously protected. Each participant was assigned an anonymized study ID number used for all analytical purposes. Identifiable documents, including consent forms, screening logs, and contact information, were stored separately in locked cabinets accessible only to authorized members of the study team. Electronic files were encrypted and backed up regularly on secure institutional servers.

## Efficacy estimation

### Clinical assessment and study endpoints

A comprehensive clinical evaluation was conducted for all participants at baseline (screening visit) and upon completion of the 6-month intervention phase. This assessment included detailed medical history, full physical examination, review of DDs, and venous blood sampling for laboratory investigations. All clinical evaluations were performed by hepatology specialists using standardized protocols to ensure consistency across visits.

### Primary endpoint

The primary endpoint was the recurrence of DDs. Events were adjudicated by a blinded clinical assessment committee to ensure objective classification.

### Secondary endpoints: Mechanistic biomarkers

The secondary endpoints focused on serum biomarkers representing key pathophysiological pathways relevant to cirrhosis progression. Malondialdehyde (MDA) is a sensitive marker of lipid peroxidation and oxidative stress. Nuclear factor κB (NF-κB): a molecular indicator of systemic inflammatory activation. Zonulin: a regulator of tight intestinal junctions, used as a surrogate marker of intestinal permeability and indirectly reflective of portal hypertension. Lipopolysaccharide (LPS): a validated indicator of bacterial translocation from the gut lumen into systemic circulation. All biomarkers were measured using standardized ELISA-based assays according to manufacturer instructions, with intra- and inter-assay coefficients of variation maintained below 10%.

### Exploratory and supportive endpoints

Additional exploratory endpoints were included to evaluate hepatic, renal, hematologic, electrolyte, and inflammatory status: Liver function tests: ALT, AST, total bilirubin, serum albumin, and international normalized ratio (INR). Renal function parameters: serum creatinine (Sr. Cr) and blood urea nitrogen (BUN). Complete blood count (CBC): hemoglobin, RBC count, WBC count, polymorphonuclear cell (PMN) percentage, and platelet count. Serum electrolytes: sodium (Na⁺), potassium (K⁺), and calcium (Ca²⁺). Inflammatory markers: C-reactive protein (CRP) and erythrocyte sedimentation rate (ESR).

### Sample collection

Blood samples were collected from all participants at baseline and at six months post-treatment. A total of 10 mL of venous blood was drawn from the antecubital vein in the morning between 8:00 and 10:00 a.m. under standardized conditions. Standard laboratory techniques were used to assess hematologic parameters, liver and renal function, and inflammatory markers. Serum malondialdehyde (MDA) levels were measured colorimetrically using a commercial assay kit (Beijing Solarbio Science & Technology, China; Catalog No. BC0020). Serum zonulin, nuclear factor κB (NF-κB), and lipopolysaccharide (LPS) levels were quantified using enzyme-linked immunosorbent assay (ELISA) kits according to the manufacturers’ instructions (Develop, Japan; Catalog Nos. DLR-Hpt-Hu, DLR-NF-κB-Hu, and DLR-LPS-Ge, respectively).

### Diagnosis and assessment of breakthroughs of decompensation events

#### Diagnostic criteria and definition of exacerbations

The diagnosis of DDs and the grading of their severity were based on established international guidelines^2^. Ascites was diagnosed by clinical examination and confirmed by abdominal ultrasonography^12^. Hepatic encephalopathy was identified primarily based on characteristic neuropsychiatric manifestations, including disturbances in attention, personality changes, apathy, delirium, and disorientation, after excluding alternative causes of encephalopathy. A focused neurological examination assessing asterixis, constructional apraxia, and bilateral extensor plantar responses supported the diagnosis^13^. Minimal HE was evaluated using the Psychometric Hepatic Encephalopathy Score (PHES), which assesses attention, visual perception, and psychomotor speed^14^. Spontaneous bacterial peritonitis was diagnosed via diagnostic paracentesis showing polymorphonuclear cell count ≥ 250 × 10⁶ cells/L, with or without positive culture^15^. Variceal bleeding was confirmed by clinical presentation and esophagogastroduodenoscopy (EGD)^16^. Hepatorenal syndrome diagnosis followed consensus criteria, distinguishing HRS-AKI (≥ 50% increase in serum creatinine within three months) from HRS-NAKI (< 50% increase within three months)^17^. Jaundice was diagnosed based on elevated total serum bilirubin levels^18^..

### Criteria for exacerbation of decompensation events

Ascites exacerbation was defined as a ≥ 1-grade increase in ascitic volume or the need for escalation of diuretic therapy^19^. The exacerbation of HE was defined as a ≥ 1-grade worsening in the West Haven/Conn score from baseline, with or without changes in asterixis^20^. Spontaneous bacterial peritonitis exacerbation was determined by either a new positive ascitic fluid culture or an increase in PMN count to ≥ 250 cells/mm³^21^.Variceal bleeding exacerbation was based on new clinical bleeding episodes confirmed by EGD^22^. Jaundice exacerbation was defined as total bilirubin > 5 mg/dL^18^.

### Adverse event monitoring

Adverse events (AEs) were actively monitored throughout the study period through patient self-reporting, structured clinical evaluations, and serial laboratory tests of liver and renal function. All AEs and serious adverse events (SAEs) were documented in accordance with Good Clinical Practice standards.

### Statistical analysis

#### Sample size determination and statistical analysis

The primary endpoint for the sample-size calculation was the recurrence of decompensation events (DDs). Assuming a 30% event rate in the control group and aiming to detect an absolute risk reduction of 25% points, with a two-sided α = 0.05 and a power of 80% (1 − β = 0.80), the required sample size was estimated using the standard normal approximation for comparing two proportions. This calculation yielded a minimum of 44 participants per study arm (88 participants in total). To account for anticipated drop-out and loss to follow-up, the target enrolment was increased to 100 participants, ensuring adequate statistical power to detect the prespecified absolute difference between groups.

The formula applied was:

$$n = \frac{{x_1^2 - \alpha ,df}}{{{w^2}}}w = \sqrt {\sum\limits_{i = 1}^k {\frac{{{{({p_i} - \overline p )}^2}}}{{\overline p }}} }$$

n = total sample size required.

α = type I error rate (0.05).

df = k − 1 (degrees of freedom).

χ² (1 − α, df) = critical value from the chi-square distribution.

p̄ = mean of the proportions across groups.

Missing data for continuous variables were handled using the last observation carried forward (LOCF) method, whereas time-to-event outcomes were analysed using censoring at the last available follow-up. Efficacy analyses were conducted on the per-protocol population, which included all participants who completed the full treatment period and all scheduled follow-up assessments. Normality of continuous variables was assessed using the Shapiro–Wilk test. Continuous variables were summarized as mean ± standard deviation (SD), while categorical variables were presented as counts and percentages. Comparisons between groups were performed using the Chi-square test for categorical variables and Student’s t-test for normally distributed continuous variables. Time-to-event outcomes were evaluated using the Kaplan–Meier method to estimate the cumulative incidence of decompensation events throughout the study period, and follow-up time for each patient was considered from randomization until occurrence of an event or censoring at the end of the study, allowing inclusion of all available data and appropriately accounting for varying follow-up durations. Differences between treatment groups in the time to first complication were assessed using Cox proportional hazards models with two-sided significance testing. All statistical analyses were performed using SPSS version 22.0 (IBM, USA). A p-value < 0.05 was considered statistically significant.

## Results

This study was conducted between June 2022 and February 2023. A total of 110 patients met the inclusion criteria and were enrolled in the study. Patients who did not meet the inclusion criteria or met exclusion criteria (*n* = 106) were excluded due to the absence of a prior decompensation event, incomplete clinical or laboratory data, refusal to participate, severe chronic extrahepatic comorbidities, age above the upper limit, history of liver transplantation, and lactation.

**Fig. 1:**
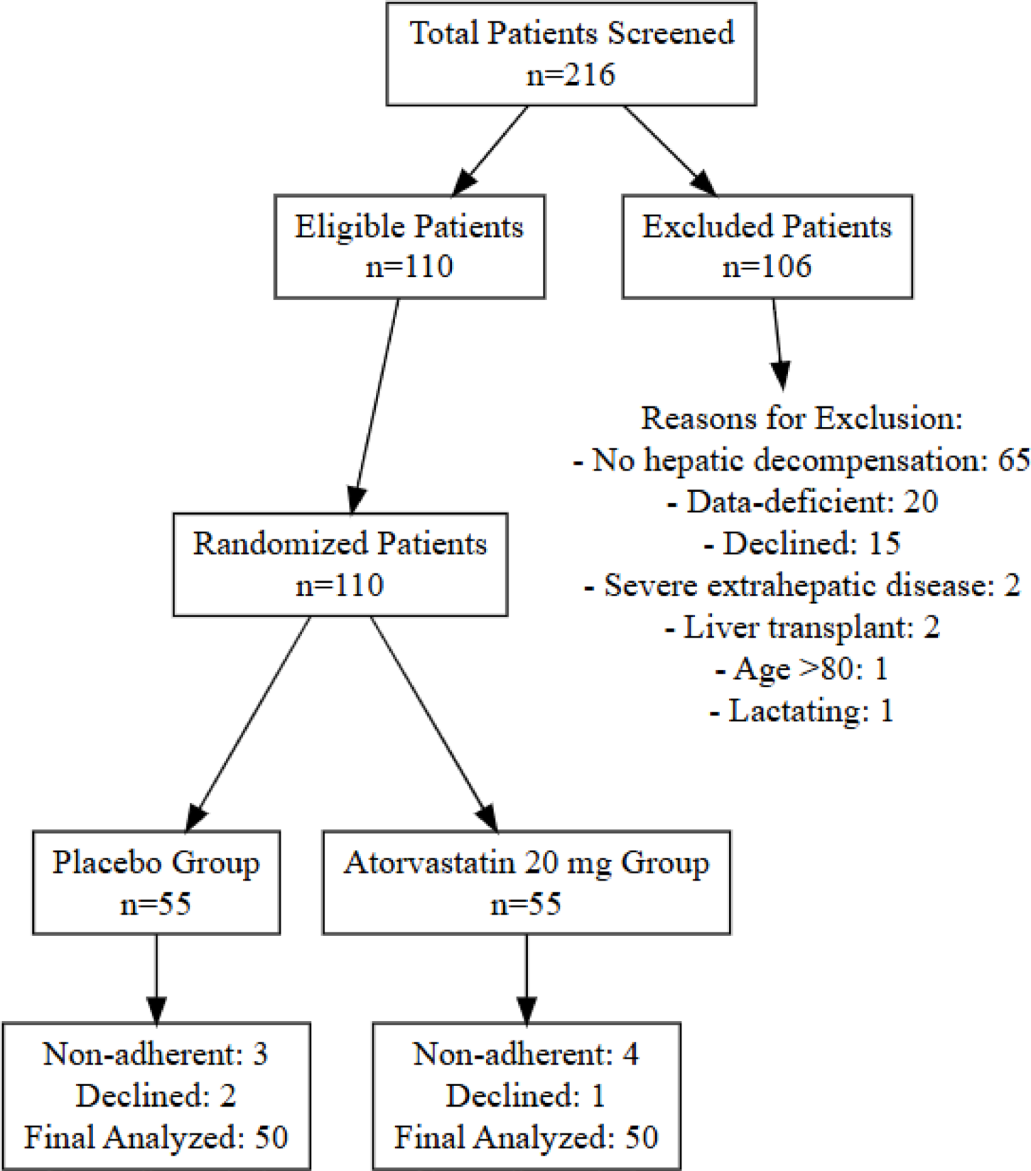
Patients’ flowchart.

Following randomization of the 110 eligible participants into the two study arms, seven individuals were excluded due to non-adherence, and an additional three patients withdrew consent. Consequently, 100 participants completed the study and were included in the final per-protocol analysis. Figure 1.

The baseline demographic and clinical characteristics of the study population were comparable between the atorvastatin and placebo groups. There were no statistically significant differences in sex distribution, age, or BMI between the two groups (*P* > 0.05). Approximately two-thirds of the participants were female, and the mean age was around 57 years. Regarding the underlying etiology of cirrhosis, viral hepatitis (HCV and HBV) represented the predominant cause in both groups, with no significant difference in distribution (*P* = 0.991). The mean disease duration was about 10 years, which was comparable between the atorvastatin and placebo arms (*P* = 0.954). Liver disease severity indices were also balanced across groups. Both the Child–Pugh score (*P* = 0.348) and MELD-Na (*P* = 0.921) showed no significant differences. Similarly, the distribution of Child–Pugh classes B and C did not differ significantly between the groups (*P* = 0.307). A comparable proportion of patients reported prior cirrhosis-related complications, including ascites, jaundice, spontaneous bacterial peritonitis, hepatic encephalopathy, variceal bleeding, and hepatorenal syndrome, with no statistically significant group differences (*P* > 0.05). Additionally, beta-blocker use was similar between groups (*P* = 0.841). Overall, these findings confirm that the two study groups were well matched at baseline, ensuring the internal validity of subsequent comparisons. Table 1.

**Table 1:** Baseline characteristics of the patients, according to study group

Characteristics	Placebo	Atorvastatin	p value
Sex	17 (34%)	18 (36%)	0.835
Male	33 (66%)	32 (64%)	
Female			
Age–years	57.14 ± 10.14	56.52 ± 9.31	0.748
BMI (Kg/m^2^)	28.75 ± 2.47	28.64 ± 2.83	0.823
Primary etiology–no. (%)	26 (52%)	26 (52%)	0.991
HCV	22 (44%)	21 (42%)	
HBV	1 (2%)	1(2%)	
Schistosoma	1(2%)	1(2%)	
Combined etiology	0 (0%)	1(2%)	
Hemochromatosis			
Course of disease (months)	129.6 ± 104	128.+64 ± 60.48	0.954
Child–Pugh score	9.5 ± 2.7	10 ± 2.6	0.348
Child-Pugh Class	33 (66%)	27 (54%)	0.307
B	17 (34%)	23 (46%)	
C			
MELD-Na^+^	14.99 ± 6.8	15.12 ± 6.14	0.921
Patients with a history of complications–no. (%)	27 (54%)	29 (58%)	0.841
Ascites	4 (8%)	6 (12%)	0.739
Grade I	20 (40%)	22 (44%)	0.841
Grade II	3 (6%)	1 (2%)	0.617
Grade III	20 (40%)	21 (42%)	1
Jaundice	12 (24%)	12 (24%)	1
SBP	13 (26%)	10 (20%)	0.629
HE	12 (24%)	15 (30%)	0.655
VB	5 (10%)	7 (14%)	0.755
HRS			
Beta blockers users (%)	21(42%)	23 (46%)	0.841

Numerical data expressed as mean ± standard deviation; nominal data expressed as number (percent) or ratio; BMI: body mass index; HCV: hepatitis C virus; HBV: hepatitis B virus; SBP: spontaneous bacterial peritonitis; HE: hepatic encephalopathy; VB: variceal bleeding; HRS: hepato-renal syndrome.

P values˂ 0.05 are considered clinically significant.

### Primary outcome assessment

#### Recurrence of liver cirrhosis–related complications

Overall, patients in the atorvastatin group experienced a significantly lower rate of cirrhosis-related complications compared with the placebo group (36% vs. 72%, *P* < 0.001), representing a 50% relative risk reduction, with an absolute risk reduction of 36% and a number needed to treat of approximately 3.

When individual complications were analyzed, atorvastatin was associated with a significant reduction in the recurrence of hepatorenal syndrome (HRS) compared with placebo (0% vs. 20%, *P* = 0.033), corresponding to a complete (100%) relative risk reduction, an absolute risk reduction of 10%, and a number needed to treat approximately 5.

All HRS cases observed in the placebo group were Type 2.

There was a trend toward a reduction in the incidence of variceal bleeding in the atorvastatin group compared with placebo (0% vs. 12%, *P* = 0.078), corresponding to an absolute risk reduction of 12% and a number needed to treat of approximately 9.

No significant differences were observed between the groups for ascites, spontaneous bacterial peritonitis (SBP), HE, or jaundice. Table 2.

**Table 2:** Comparison of the recurrence of liver cirrhosis-related complications between the two groups within the treatment period.

Complication	Placebo (*N* = 50)	Atorvastatin (*N* = 50)	Hazard ratio (95%CI)	*P* value	ARR	NNT
All cirrhosis-related complications	36 (72%)	18 (36%)	0.5 (0.33–0.75)	< 0.001	0.36	2.78
Ascites	12 (24%)	10 (20%)	0.83 (0.39–1.75)	0.63	0.04	25
SBP	4 (8%)	2 (4%)	0.5 (0.096–2.61)	0.41	0.04	25
Jaundice	5 (10%)	5 (10%)	1 (0.31–3.24)	1	---	---
HE	11(22%)	8 (16%)	0.73 (0.32–1.65)	0.45	0.06	16.67
VB	6 (12%)	0 (0%)	0.077 (0.0044–1.33.0044.33)	0.078	0.12	8.5
HRS	10 (20%)	0 (0%)	0.047 (0.003–0.79)	0.033	0.2	5.1

Data is expressed as number (percentage); SBP: Spontaneous bacterial peritonitis; HE: Hepatic encephalopathy; VB: Variceal bleeding; HRS: Hepatorenal syndrome; ARR.

:absolute risk reduction; NNT: number needed to treat.

Statistically significant (*p* < 0.05).

### Secondary outcomes assessment

#### Hepatic decompensation-related biomarkers

The effects of atorvastatin on hepatic decompensation–related biomarkers over the 6-month follow-up are presented in Table 3. At baseline, levels of malondialdehyde (MDA), NF-κB, lipopolysaccharide (LPS), and zonulin were comparable between the placebo and atorvastatin groups, with not statistically significant between-group differences (all P1 > 0.05).

**Table 3:** Hepatic decompensation-related biomarkers

Parameter	Placebo (N=50)	Atorvastatin (N=50)	P1-value
MDA (mg/dl)			
Baseline	49.25±13.08	47.66±14.26	0.835
After 6 months	45.75±9.38	30.07±13.86	<0.001^*^
P2	0.06	˂ 0.001^*^	
NFκB (ng/ml)			
Baseline	12.12±3.74	12.19±3.92	0.994
After 6 months	13.1±6.02	6.84±1.83	<0.001^*^
P2	0.292	˂ 0.001^*^	
Lipopolysaccharide (ng/ml)			
Baseline	102.2±42.7	103.16±44.82	0.965
After 6 months	114.95±54.23	67.6±35.93	<0.001^*^
P2	0.17	˂0.001^*^	
Zonulin (ng/ml)			
Baseline	7.82±3.05	7.92±2.91	0.855
After 6 months	7.36±3.37	3.19±1.63	<0.001^*^
P2	0.446	˂0.001^*^	

Data are presented as mean ±SD.

MDA: malondialdehyde; NFκB: nuclear factor kappa B; P_1_-value: Between groups statistical difference; P_2_-value; Within group statistical difference; a: p-value between placebo and allopurinol; b: p-value between placebo and atorvastatin.

*Statistically significant (p <0.05)

After 6 months of treatment, the atorvastatin group showed a statistically significant decline in all assessed biomarkers compared with baseline (all P2 < 0.001). In contrast, no significant within-group changes were observed in the placebo group over the same period (all P2 > 0.05). Between-group analysis at 6 months demonstrated significantly lower biomarker levels in the atorvastatin group than in the placebo group (all P1 < 0.001 for MDA, NF-κB, LPS, and zonulin).

#### Hematological and Liver Biochemistry Parameters

Hematological parameters were comparable between the placebo and atorvastatin groups at baseline, with no significant differences observed in hemoglobin, red blood cell count, white blood cell count, polymorphonuclear cell count, or platelet count (all P1 > 0.05).

Over the 6-month follow-up period, no significant within-group changes were detected in hemoglobin, red blood cell count, white blood cell count, or platelet count in either group (all P2 > 0.05). However, a statistically significant reduction in polymorphonuclear (PMN) cell count was observed in the atorvastatin group (P2 < 0.001).

There were no statistically significant differences between the placebo and atorvastatin groups at baseline for ALT, AST, total bilirubin, albumin, or INR (all *P* > 0.05). In addition, in the placebo group, no significant changes were observed after 6 months as compared to their baselines.

In contrast, the atorvastatin group demonstrated significant within-group increases after 6 months in ALT (*P* < 0.001), AST (*P* < 0.001), total bilirubin (*P* < 0.001), and albumin (*P* = 0.008), while INR did not change significantly. Between-group comparisons after 6 months showed significantly higher ALT, AST, total bilirubin, and albumin levels in the atorvastatin group compared with placebo (all *P* < 0.001), whereas INR remained comparable between groups. Table 4.

**Table 4:** Data of hematological test and liver enzyme test.

Parameter	Placebo	Atorvastatin	P1-value
HB(g/dl)			
Baseline	10.46±1.04	10.36±.1.09	0.64
After 6 months	10.56±1.19	10.57±1.01	0.964
P2	0.615	0.138	
RBCs(10^6^U/L)			
Baseline	3.93±0.87	3.89±.0.86	0.818
After 6 months	3.9±0.89	3.79±0.83	0.524
P2	0.879	0.526	
WBCs(10^3^U/L)			
Baseline	5.95±1.56	6.14±01.4	0.522
After 6 months	6.1±1.25	6.09±0.13	0.955
P2	0.653	0.86	
PMNs(10^3^U/L)			
Baseline	76.36±8.54	76.08±7.08	0.859
After 6 months	76.04±7.63	62.04±12.52	<0.001
P2	0.844	<0.001	
PLTs(10^3^U/L)			
Baseline	165±14.2	164.88.±13.76	0.966
After 6 months	165±13.46	161.78±11.6	0.203
P2	1	0.226	
ALT(U/L)			
Baseline	25.1±16.21	26.54±9.17	0.586
After 6 months	27.98±8.62	32.42±9.02	<0.001
P2	0.172	<0.001	
AST(U/L)			
Baseline	41.4±19.7	40.14±10.83	0.693
After 6 months	39.78±10.44	49.7±12.46	<0.001
P2	0.595	<0.001	
T-BIL (mg/dl)			
Baseline	2.66±2.61	3.22±1.98	0.852
After 6 months	2.57±2.45	3.95±2.21	<0.001
P2	0.266	<0.001	
ALB(g/dl)			
Baseline	2.92±0.63	2.94±0.42	0.852
After 6 months	2.97±0.51	3.22±0.41	0.008
P2	0.097	<0.001	
INR			
Baseline	1.45±0.37	1.42±0.38	0.69
After 6 months	1.53±0.53	1.56±0.53	0.778
P2	0.7	0.138	

Data are presented as mean ±SD; Hb: Hemoglobin; RBCs: Red Blood Cells; WBCs: White Blood Cells; PLTs: platelets; ALT: Alanine aminotransferase; AST : Aspartate transaminase; T-BIL: Total bilirubin; ALB: albumin; INR: international normalized ratio; P_1_-value: Between groups statistical difference; P_2_-value;:Within group statistical difference

Statistically significant (p <0.05)

#### Renal function, serum electrolytes, and oxidative/inflammatory biomarkers

Renal function parameters demonstrated clear differences between the two study groups after 6 months of treatment. Serum creatinine and BUN levels remained stable in the placebo group (*p* = 0.206 and *p* = 0.871, respectively). In contrast, patients treated with atorvastatin exhibited significant decreases in both creatinine and BUN (*p* < 0.001 for both) compared with baseline. These changes translated into significant between-group differences at 6 months for both S.Cr and BUN (*p* < 0.001 for each).

Serum electrolytes (Na⁺, K⁺, and Ca⁺⁺) did not show significant differences between the two groups at baseline or at study completion (all *p* > 0.05). Likewise, within-group changes over time were minimal and non-significant across both treatment arms, confirming that atorvastatin had no measurable impact on electrolyte balance.

Inflammatory biomarkers demonstrated a marked response to atorvastatin therapy. CRP levels decreased significantly in both groups, though the magnitude of reduction was substantially greater in the atorvastatin group (*p* < 0.001), resulting in a highly significant between-group difference at 6 months (*p* < 0.001). ESR1 and ESR2 also showed significant within-group reductions in both arms; however, a significant between-group difference was observed only for ESR2 at the end of treatment (*p* = 0.042), favouring atorvastatin. Table 5.

**Table 5:** Data of renal function test, serum electrolytes, and serum malondialdehyde

Parameter	Placebo (N=50)	Atorvastatin	P1-value
S.cr			
Baseline	1.06±.402	1.1±0.37	0.586
After 6 months	1.16±.43	0.83±0.25	0
P2	0.206	0	
BUN (mg/dl)			
Baseline	38.3±14.27	37.38±10.93	0.733
After 6 months	38.74±12.9	28.98±9.11	0
P2	0.871	0	
Na^+^ (mEq/l)			
Baseline	134.85±4.5	136.05±4.16	0.158
After 6 months	134.59±3.9	134.86±4.33	0.745
P2	0.772	0.202	
K^+^ (mEq/l)			
Baseline	3.74±0.59	3.9±0.58	0.168
After 6 months	3.65±0.69	3.8±0.55	0.262
P2	0.465	0.354	
Ca^++^ (mg/dl)			
Baseline	1.13±0.07	1.1±0.2	0.069
After 6 months	1.12±0.09	1.13±0.12	0.271
P2	0.519	0.145	
CRP (mg/dl)			
Baseline	53.06±24.8	48.76±14.32	0.291
After 6 months	34.92±10.18	15.86±6.05	<0.001
P2	0.011	<0.001	
ESR1			
Baseline	48.3±18.02	49.08±18.05	0.0.829
After 6 months	44.74±18.24	40.68±15.52	0.234
P2	0.027	<0.001	
ESR2			
Baseline	96±36.05	96.16±27.35	0.945
After 6 months	89±36.48	76.6±25	0.042
P2	0.027	<0.001	

Data are presented as mean ±SD; S.Cr: Serum creatinine; BUN: blood urea nitrogen

P_1_-value: Between groups statistical difference; P_2_-value;:Within group statistical difference

Statistically significant (p <0.05)

### Reported side effects during the treatment period

Throughout the treatment period. Overall, most side effects were mild and comparable between the two groups. Skin reactions (rash) were observed in 10% of patients receiving atorvastatin, while none were reported in the placebo group (*P* = 0.081). Gastrointestinal symptoms—including nausea, diarrhoea, stomach pain, constipation, and heartburn—occurred at similar rates in both groups, with no statistically significant differences (*P* > 0.05 for all). Neurological symptoms, including dizziness, drowsiness, and headache, were also comparable between groups.

Among musculoskeletal symptoms, the incidence of muscle pain/myalgia was significantly higher in the atorvastatin group compared to placebo (14% vs. 2%; *P* = 0.036), whereas joint pain/arthralgia was similar between groups (8% in both, *P* = 0.73).

These results indicate that atorvastatin was generally well tolerated, with the main notable side effect being a higher incidence of myalgia, while other adverse events did not differ significantly from placebo. Table 6..

**Table 6:** Reported side effects for patients in the studied groups during the treatment period

Side effect	Placebo (N=50)	Atorvastatin (N=50)	P-value
Skin reaction (Rash)	0 (0%)	5 (10%)	0.081
GIT symptoms			
Nausea	2 (4%)	3(6%)	0.701
Diarrhea	1 (2%)	2(4%)	0.594
Stomach Pain	4 (8%)	1(4%)	0.248
Constipation	2 (4%)	5 (10%)	0.18
Heartburn	3(6%)	4 (8%)	0.397
Neurological symptoms			
Dizziness	1 (2%)	6 (12%)	0.155
Drowsiness	9 (18%)	8 (16%)	0.698
Headache	6 (12%)	7 (14%)	0.827
Musculoskeletal symptoms			
Joint Pain/ Arthralgia	4 (8%)	4 (8%)	0.73
Muscle Pain/Myalgia	1 (2%)	7 (14%)	0.036^*^

Data expressed as a number (percentage) and tested by the Chi-square test.

GIT: Gastrointestinal tract

^*^Statistically significant (P1 <0.05)

## Discussion

Statins are often under-prescribed in patients with cirrhosis, even when clear indications such as dyslipidemia or prior cardiovascular events exist^23^. Beyond their established role in preventing atherosclerotic cardiovascular disease, statins have been shown to exert pleiotropic effects, providing additional benefits in conditions including chronic obstructive pulmonary disease, acute kidney injury, pancreatitis, and erectile dysfunction^24^..

Emerging evidence indicates that statins may provide benefits in both the primary and secondary prevention of cirrhosis, shifting their perception in chronic liver disease from risky to potentially beneficial^25–27^. Previous studies have shown that statins can slow hepatic fibrosis, prevent decompensation, and reduce all-cause mortality in patients with chronic liver disease^28,29^..

Given the limited evidence, further studies are needed before routine use in cirrhosis. In this context, we conducted a double-blind, placebo-controlled trial to evaluate the efficacy of atorvastatin in preventing hepatic decompensation. Our results demonstrated that atorvastatin administration led to a 50% reduction in overall cirrhosis-related complications, with the largest decreases observed in hepatorenal syndrome and a trend toward reduction in variceal bleeding. These findings align with previous reports indicating that statins have beneficial effects in cirrhosis^3,23,30^. Collectively, studies suggest that statins are safe in chronic liver disease, can modulate hemostasis through hepatic, endothelial, and platelet mechanisms, may reduce portal hypertension, lower the risk of decompensation and variceal bleeding, and potentially improve overall clinical outcomes, including mortality, particularly in patients with compensated cirrhosis.

Serum malondialdehyde (MDA), a marker of oxidative stress, was markedly elevated in cirrhotic patients at baseline, consistent with previous studies highlighting oxidative stress as a hallmark of cirrhosis^31^. In our study, atorvastatin administration significantly reduced serum MDA levels, supporting prior evidence that statins can mitigate oxidative stress and decrease inflammatory cell activation^32^..

Zonulin, a precursor of haptoglobin-2, is released in response to intestinal epithelial damage from bacteria or portal hypertension, disrupting tight junctions and increasing gut permeability^33,34^. Serum zonulin thus serves as a biomarker of intestinal barrier dysfunction and predicts hepatic decompensation. In our study, atorvastatin significantly reduced zonulin levels, indicating a protective effect against decompensation events.

Lipopolysaccharide (LPS), a marker of bacterial translocation, is associated with systemic inflammation and hemodynamic instability^35^. In our study, atorvastatin reduced circulating LPS, consistent with previous reports^10^..

LPS triggers Toll-like receptor 4 (TLR4) activation, leading to nuclear translocation of NF-κB and transcription of pro-inflammatory cytokines such as TNF-α, MCP-1, IL-6, and pro-IL-1β, which promote apoptotic and necroptotic pathways^36^. NF-κB thus serves as a reliable indicator of inflammatory and immune responses in decompensated cirrhosis.

In the present study, atorvastatin administration was associated with a reduction in NF-κB levels, accompanied by decreased systemic inflammation and the serum levels of CRP and ESR, and these matches finding reported by Planavil work^37^..

Hepatic decompensation arises from oxidative stress, systemic inflammation, gut barrier dysfunction, and bacterial translocation, all contributing to recurrent cirrhosis-related complications. In our study, atorvastatin reduced overall decompensation events by 50%, accompanied by significant decreases in key biomarkers: MDA (oxidative stress), zonulin (intestinal barrier integrity), LPS (bacterial translocation), and NF-κB (pro-inflammatory signaling). These findings underscore the pleiotropic effects of atorvastatin in modulating hepatointestinal and systemic homeostasis, providing a pathophysiological basis for its protective role, consistent with prior evidence of statins’ safety and beneficial effects on portal hypertension, systemic inflammation, and clinical outcomes in compensated cirrhosis.

Although atorvastatin reduced overall cirrhosis-related complications, no significant differences were observed for ascites, SBP, HE, or jaundice, with the most pronounced benefits seen in HRS and a trend toward reduction in variceal bleeding.

Several physiological and temporal factors may explain the selective impact of atorvastatin on specific complications. HRS-2 develops gradually in patients with refractory ascites, driven by progressive renal dysfunction, systemic inflammation, and endothelial dysregulation^38^. Epidemiological evidence indicates that HRS often occurs over months to years, rather than in the short-term recurrence of ascites^39^..

Accordingly, the six-month duration of our trial may have been sufficient to capture the protective effects of atorvastatin on the slowly evolving HRS-2, while being too short to detect statistically significant differences in other multifactorial decompensation events such as ascites, The six-month duration of our trial may have been sufficient to capture atorvastatin’s protective effects on slowly evolving HRS-2, while being too short to detect significant differences in other multifactorial complications such as ascites, SBP, HE, or jaundice. Mechanistically, atorvastatin may selectively modulate endothelial function, oxidative stress, systemic inflammation, and intrarenal signaling, interrupting the pathophysiological cascade leading to HRS-2 without preventing short-term recurrence of other complications^40^. We acknowledge that the limited number of complication-specific events and follow-up duration are important limitations, and these findings should be considered exploratory. Larger, longer-term studies are needed to confirm these results and clarify the reno-protective role of atorvastatin in cirrhosis.

Although chronic kidney disease (CKD) and HRS are distinct, they share key mechanisms, including systemic inflammation, endothelial dysfunction, and oxidative stress. Evidence from CKD shows that atorvastatin can improve renal function, reduce proteinuria, and modulate inflammatory and oxidative pathways^41^. By extrapolation, these pleiotropic effects may partly explain atorvastatin’s protective impact on HRS.

Our findings show some discrepancies compared to the StatLiver trial^42^. These differences may reflect our single-center, more homogeneous population predominantly with HBV- and HCV-related cirrhosis, variations in baseline disease severity, concomitant medications, adherence to standard therapy, smaller sample size, and differences in outcome definitions and follow-up protocols. Collectively, these factors indicate that, although promising, our results should be interpreted cautiously and considered hypothesis-generating until confirmed in larger, multicenter trials.

Concerning atorvastatin safety, atorvastatin treatment has been associated with a mild elevation in liver enzymes. In LiverTox, it was noted that atorvastatin therapy is associated with minor, asymptomatic, and typically transient elevations in serum aminotransferase levels^43^. However, the reported mild increase in liver enzymes in our study in the atorvastatin group was not of clinical significance according to HY’s law^44^..

Several limitations of this study warrant consideration. First, the absence of hepatic venous pressure gradient (HVPG) measurements, the gold standard for quantifying portal hypertension, precludes a direct assessment of atorvastatin’s hemodynamic effects. While HVPG provides critical mechanistic insights, its invasive nature and requirement for specialized interventional expertise often limit its feasibility in broader clinical settings. To address this, we prioritized patient-centred, clinically hard endpoints, including variceal bleeding recurrence and hepatorenal syndrome. These outcomes offer a pragmatic reflection of the drug’s impact on the natural history of cirrhosis. Furthermore, the observed improvements in biomarkers of bacterial translocation (LPS, zonulin) and systemic inflammation (NF-κB) provide an indirect yet biologically plausible link between atorvastatin therapy and reduced portal pressure, likely mediated by improved intrahepatic microcirculation. Second, the current research is limited by the lack of repeated measurements for laboratory biomarkers throughout the study period. Rather than assessments being performed at baseline and at the end of the 6-month follow-up. This restricted our ability to capture potential dynamic changes over time. It also lacked the measurement of creatinine kinase as a marker for statin-induced rhabdomyolysis. In addition, the single-centre design and the inclusion of only Egyptian patients, with viral hepatitis as the predominant etiology of cirrhosis, may limit the generalizability of the findings to other populations and to cirrhosis of different etiologies. Finally, the number of prior decompensation episodes before enrolment was not consistently available for all patients and therefore could not be analysed, which may represent a potential source of residual confounding. While the double -blind design minimizes bias, the magnitude of the clinical benefit observed here requires validation in larger, multi-ethnic, multicentre cohorts. Future investigations should incorporate serial HVPG measurements and long-term survival analysis to fully elucidate the hemodynamic mechanisms and the durability of the protective effects observed in this trial.

## Conclusion

This randomized, double -blind, placebo-controlled trial suggests that a six-month course of atorvastatin (20 mg/day) is associated with a lower risk of recurrent decompensation in patients with advanced cirrhosis. Treatment with atorvastatin was also associated with a lower incidence of hepatorenal syndrome and a numerical reduction in variceal bleeding events. These clinical findings were accompanied by changes in biomarkers related to oxidative stress, systemic inflammation, and intestinal barrier function. While these observations are consistent with potential pleiotropic effects of atorvastatin, including modulation of the gut–liver axis, causal mechanisms cannot be established from the present study. Atorvastatin was generally well tolerated; transient elevations in transaminases were not clinically relevant, although mild myalgia occurred more frequently and warrants monitoring. Overall, these findings indicate a possible adjunctive role for atorvastatin in decompensated cirrhosis, which requires confirmation in larger, multicenter trials with dedicated mechanistic and hemodynamic assessments.

## Supplementary Information

Below is the link to the electronic supplementary material.

Supplementary Material 1

## Data Availability

The full study protocol and statistical analysis (De-identified participant data and statistical code) plan will be made available from the corresponding author upon reasonable request.
